# Clinical and economic burden of pneumococcal disease among individuals aged 16 years and older in Germany

**DOI:** 10.1017/S0950268822001182

**Published:** 2022-11-08

**Authors:** Arijita Deb, Bélène Podmore, Rosemarie Barnett, Dominik Beier, Wolfgang Galetzka, Nawab Qizilbash, Dennis Haeckl, Sarah Mihm, Kelly D. Johnson, Thomas Weiss

**Affiliations:** 1 Merck & Co., Inc., Rahway, NJ, USA; 2 OXON Epidemiology, London, UK; 3 London School of Hygiene & Tropical Medicine, London, UK; 4 University of Bath, Bath, UK; 5 InGef – Institute for Applied Health Research Berlin GmbH, Berlin, Germany; 6 WIG2 GmbH, Leipzig, Germany; 7 MSD Sharp & Dohme GmbH, Munich, Germany

**Keywords:** All-cause pneumonia, claims data, Germany, healthcare resource utilisation, invasive pneumococcal disease

## Abstract

This study assessed the incidence rate of all-cause pneumonia (ACP) and invasive pneumococcal disease (IPD) and associated medical costs among individuals aged ≥16 in the German InGef database from 2016 to 2019. Incidence rate was expressed as the number of episodes per 100 000 person-years (PY). Healthcare resource utilisation was investigated by age group and by risk group (healthy, at-risk, high-risk). Direct medical costs per ACP/IPD episode were estimated as the total costs of all inpatient and outpatient visits. The overall incidence rate of ACP was 1345 (95% CI 1339–1352) and 8.25 (95% CI 7.76–8.77) per 100 000 PY for IPD. For both ACP and IPD, incidence rates increased with age and were higher in the high-risk and at-risk groups, in comparison to the healthy group. ACP inpatient admission rate increased with age but remained steady across age-groups for IPD. The mean direct medical costs per episode were €8075 (95% CI 7121–9028) for IPD and €1454 (95% CI 1426–1482) for ACP. The aggregate direct medical costs for IPD and ACP episodes were estimated to be €8.5 million and €248.9 million respectively. The clinical and economic burden of IPD and ACP among German adults is substantial regardless of age.

## Introduction

Pneumococcal infections are a major cause of communicable disease morbidity and mortality in children and the elderly worldwide [1]. Globally, over 1.6 million die annually as a result of pneumococcal disease (PD) [1]. PD can be grouped into non-invasive and invasive disease, ranging from mild respiratory tract mucosal infections to severe invasive disease. The more serious clinical manifestations of PD are pneumonia, bacteraemia and meningitis, with pneumococcal pneumonia the most common clinical presentation of PD among adults [2]. Bacteraemia and meningitis are collectively referred to as invasive pneumococcal disease (IPD). IPD can also include other severe infections such as osteomyelitis or septic arthritis. PD affects mostly children, adults over the age of 50 and individuals with additional comorbidities and risk-factors, for example, those with chronic conditions such as chronic heart, liver, kidney or lung disease [1, 3–5].

Pneumococcal infections and IPDs are major causes of mortality in Europe, with hospital mortality estimated at 15% [6]. Despite the implementation of various widespread vaccination programs against PD since the 1980s, the clinical burden of PD throughout Europe remains substantial [4, 5, 7, 8].

A review of historic data from across Europe suggests that pneumonia overall may directly cost approximately €10.1 billion annually [4]. Specifically, IPD has been identified as a major cause of PD-related hospitalisation and treatment costs in European countries [9]. With an aging European population, these costs are predicted to further increase in future [8, 10, 11]. The global prevalence of PD is also evolving due to the changing distribution of pneumococcal serotypes, in particular an increase in non-vaccinated serotypes [12–15]. Continued surveillance of the clinical and economic burden of PD and serotype distribution is therefore critical, to determine residual/ changing burden of disease and refine pneumococcal vaccination schedules.

In Germany, vaccination recommendations are reviewed yearly by the German Standing Committee on Vaccination (Ständige Impfkommission, STIKO) and are published in the epidemiological bulletin of the Robert Koch Institute [16, 17]. Since 1998, the 23-valent pneumococcal polysaccharide vaccine (PPSV23) has been recommended as standard pneumococcal vaccination for all individuals aged 60 and over [18]. Universal vaccination with pneumococcal conjugate vaccine (PCV) is recommended in a 2 + 1 schedule for children up to 2 years old. Furthermore, pneumococcal vaccination is recommended for children >2 years, adolescents and adults with underlying chronic conditions. Until 2010, PPSV23 was the only pneumococcal vaccine licensed for adults in Germany. However, in 2011, the European Medicines Agency licenced the 13-valent pneumococcal conjugate vaccine (PCV13) for use in adults aged 50 years and older [19]. STIKO now recommends sequential vaccination with PCV13 followed by PPSV23 after 6–12 months in individuals aged >2 with an underlying medical condition with ‘high-risk’ of PD, due to congenital or acquired immunodeficiencies or immunosuppression, and for children aged 2–15 ‘at-risk’ of PD due to chronic conditions such as cardiovascular, metabolic or respiratory disease [16, 17, 20]. For ‘at-risk’ individuals aged 16 and over, PPSV23 is recommended. Sequential vaccination is not recommended for standard vaccination in older adults due to the low number of additional preventable cases and the very high number needed to vaccinate [21].

A previous study conducted in Germany using the Institute for Applied Health Research (InGef) database estimated incidence rates of all-cause pneumonia (ACP) in individuals aged ≥18 years between 2008–2012 in Germany [7]. That study found that incidence of pneumonia increased with increasing age and among individuals with comorbid conditions. However, recent studies on pneumococcal disease incidence in Germany are lacking. Studies investigating fatality rates, healthcare resource utilisation (HCRU), costs, time and age trends are also limited. In the present study, we therefore aimed to provide updated estimates of the clinical and economic burden of ACP and IPD in adults in Germany, utilising data from a large claims healthcare database.

## Materials and methods

### Study design

This was a retrospective cohort study of individuals aged ≥16 years in Germany, utilising the InGef (Institute for Applied Health Research Berlin, formerly Health Risk Institute) research database. The InGef database is comprised of de-identified longitudinal claims data from more than 9 million individuals across more than 70 statutory health insurance providers (SHIs) throughout Germany [22]. The present study used a sample dataset of approximately 4 million individuals who are representative of the German population with regards to age and sex [23]. The database includes demographic information (gender, age, region of residence); diagnoses data; claims data for ambulatory services and procedures in alignment with the German uniform evaluation standard (EBM,‘Einheitlicher Bewertungsmaßstab’); specific data on outpatient visits; inpatient hospital admissions (including admission and discharge dates, both primary and secondary discharge diagnoses and codes for procedures conducted in hospital according to the German Procedure Classification (OPS, ‘Operationen und Prozedurenschlüssel’)); mortality rates and morbidity data; drug prescription and dispensing data (drug name, quantity, date of prescription and dispensing); and healthcare costs [23]. All diagnoses are recorded using the 10th revision of the International Classification of Diseases German Modification (ICD-10-GM).

### Study population

The source population for this study included adults (≥16 years old) in Germany within the InGef research database between 1 January 2014 to 31 December 2019. To be eligible for inclusion in the study, all individuals had to be continually insured with one of the SHIs contributing data to the database for 24 months prior to study inclusion (study ‘pre-period’) to ensure their medical history could be assessed for presence of at-risk and high-risk conditions and pneumococcal vaccination. As such, for individuals who started contributing data after 1 January 2014, their study entry date was the date they started contributing data to the InGef research database plus 24 months. The study inclusion period therefore started on 1 January 2016 and ended 31 December 2018 (allowing all adults to have the potential for one year of follow-up data). The study observation period for adults covered 1 January 2016 to 31 December 2019, during which study outcomes could be observed.

**Fig. 1.**
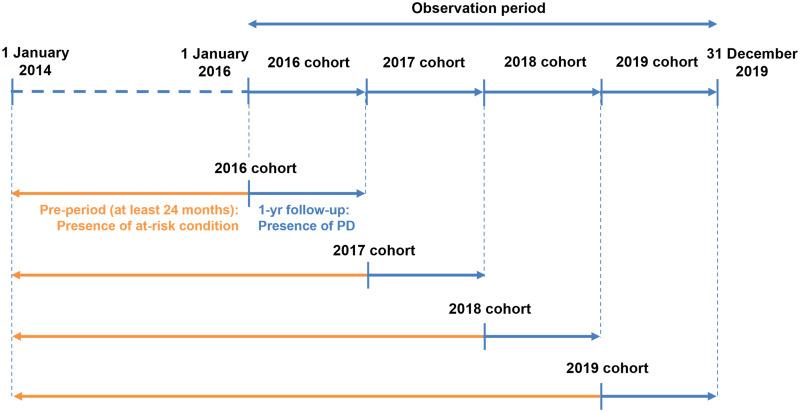
Clinical burden of PD study design.

Four yearly cohorts (Fig. 1) were created to assess the clinical and economic burden of PD within each calendar year of the study (2016–2019), in which individuals started contributing data from the latest of the following dates: start of study year (1 January), or the date they started contributing data to the InGef research database plus 24 months. For example, an individual who started contributing data on 1 June 2014 would be included in the 2016 cohort from 1 June 2016 and included in the 2017, 2018 and 2019 cohorts from 1 January of the respective study years.

During each study year, each individual was followed-up from the start of the calendar year (1 January) until the first of the following censoring criteria: end of observation in the InGef research database (based on: end of insurance with SHI contributing data to the InGef research database, end of the study year (31 December), end of study period (31 December 2019), death from any cause), or date of first pneumococcal vaccination received during the observation period. Individuals who received pneumococcal vaccination during the observation period were excluded from the following yearly cohorts. For example, those who were vaccinated in 2017 were excluded from the 2018 and 2019 cohorts. This was to ensure assessment of vaccine-preventable PD i.e., incidence of ACP and IPD in the unvaccinated population.

The population was described based on underlying medical conditions linked to higher risk of pneumococcal disease according to the 2016/2017 and 2017/2018 STIKO recommendations for at-risk/high-risk individuals [16, 17]; whereby individuals were assigned to one of three groups at the start of each study year: ‘high-risk’, ‘at-risk’ and ‘healthy’ (Table 1). Individuals without any at-risk or high-risk underlying medical conditions were classified as healthy for the purpose of this study. Underlying medical conditions were also reported for the overall study population in the 24-month pre-period. The underlying medical conditions were identified by ICD-10-GM codes in the outpatient and inpatient data (all diagnosis fields) as well as OPS codes, EBM codes and ATC codes for prescriptions in the InGef research database.

**Table 1.** Risk condition classifications

Risk classification	Risk conditions
At-risk	Diabetes mellitus, chronic lung disease (including asthma), chronic heart disease and neurological disorders
High risk	Cancer, cerebrospinal fluid leak, chronic renal disease, cochlear implant, functional or anatomic asplenia, sickle cell disease/other hemoglobinopathy, congenital or acquired asplenia, splenic dysfunction, splenectomy, HIV infection, other immuno-compromising diseases, organ transplants, chronic liver disease and autoimmune disease.

### Outcomes

ACP was defined as all cases of pneumonia caused by all unknown and known pathogens and was identified in the database with codes both specifically for pneumococcal pneumonia and non-specific codes for pneumonia including ICD-10-GM J10.0, J11.0, J12–J18, but excluding any IPD (Supplementary material S1 Code lists). ACP was chosen as a proxy for pneumococcal pneumonia, as diagnostic tests to identify causative pathogen are infrequently performed in clinical practice [9]. The diagnosis and coding of pneumococcal pneumonia would therefore underestimate disease burden [24, 25]. IPD was defined as IPD cases where pneumococcus was known to have a causative role as identified from pneumococcal-specific codes. Both ACP and IPD were identified within the outpatient and inpatient data (all diagnosis fields). In the inpatient data, date of hospital admission was used to identify diagnosis dates. At least one prescription of antibiotics in the same quarter was required to validate outpatient diagnoses, as only yearly quarter of the diagnosis was available in the outpatient database. In the outpatient data, where only yearly quarter in which the diagnosis was made was available, the first record which could be related to the ACP/IPD for example antibiotic prescriptions and diagnostic tests (e.g., chest *x*-rays), was used to assign the ACP/IPD diagnosis date.

For ACP and IPD, multiple records were considered as independent episodes if they were separated by ≥90 days. Each pneumonia and IPD episode ended at the last record within the episode plus 90 days.

Fatality rates captured only deaths at hospital discharge in hospitalised cases of ACP/IPD. Out-of-hospital deaths were not included in our definition of fatality rate, as cause of death is not recorded in the InGef database and it is therefore not possible to determine whether the death occurred due to the PD.

HCRU was extracted for ACP/IPD-related outpatient visits, inpatient admissions and number of antibiotic prescriptions dispensed in the outpatient setting. ACP/IPD-related outpatient visits were assumed from dates with records which could be related to ACP/IPD. Inpatient admissions with a diagnosis of ACP/IPD were also reported, in addition to length of stay. Number of antibiotic prescriptions dispensed in the outpatient setting was identified within each ACP/IPD episode. Costs associated with each episode were also reported, including: visit costs associated with each outpatient visit relating to ACP/IPD; outpatient pharmacy cost of antibiotic treatments dispensed during each episode; costs associated with ACP/IPD-related inpatient hospital admissions (calculated using direct costs from the insurance perspective for each hospital stay where the main discharge diagnosis was ACP/IPD).

### Statistical methods

Incidence rates of ACP and IPD were reported per 100 000 person-years (PY) with 95% confidence intervals (CIs) and calculated as the number of ACP/IPD episodes divided by the sum of PY at risk, whereby time at-risk was defined as the total follow-up time minus the time with ACP or IPD, respectively. Fatality rates were reported per 100 hospitalised cases and calculated as the number of inpatient ACP/IPD deaths divided by the number of individuals with an inpatient episode of ACP/IPD. Incidence and fatality rate ratios comparing risks groups were reported and the CI calculated using the Byar Method. To assess whether incidence or fatality rates changed significantly between 2016 and 2019, Mann–Kendall linear trend tests were used.

HCRU was described for ACP and IPD based on the number and percentage of individuals with ACP or IPD who used each resource (outpatient visits, prescriptions, inpatient visits); the total amount of each resource used; and the rate per 1000 PY (with 95% CI) of each resource. Rate was calculated as: total amount of each resource used divided by the total amount of time with ACP/IPD. Length of hospital stays were reported as minimum and maximum values and mean length of stay. Total cost of each resource was described using 2019 inflation values.

Variables to describe the study population included age (16–49; 50–59; 60–69; ≥70), sex (male, female) and risk-group (as described above, healthy, at-risk, high-risk); whereby all results were reported overall and stratified by the aforementioned variables. Incidence rates for ACP were also stratified by inpatient and outpatient cases, by age and risk group.

## Results

The final study population included 3 858 335 individuals aged 16 years or older at the start of the study period, with 24 months continual insurance prior to study entry. The mean age of the study population at study entry was 47 years (range: 16–108) (Table 2). The majority of individuals were 16–49 years old (54.08%), with 17.63%, 12.11%, 16.18% of the study population in the 50–59, 60–69 and ≥70 age groups, respectively. Over half (56.45%) of the study population had a history of at least 1 medical condition associated with increased risk of PD in the pre-period. The most common comorbidities were chronic heart disease (40.87%) and chronic pulmonary disease (26.94%).

**Table 2.** Baseline characteristics of the study population

	*N* = 3 858 335	
Number of individuals included in each study year (*n*)		
2016	3 529 035	
2017	3 406 005	
2018	3 336 317	
2019	3 248 735	
Age (years) at study entry		
Mean (Minimum, maximum)	47	(16–108)
Age group (years) at study entry (*n*, %)		
16–49	2 086 526	54.08
50–59	680 237	17.63
60–69	467 343	12.11
≥70	624 229	16.18
Sex (*n*, %)		
Male	1 892 342	49.05
Female	1 965 993	50.95
Presence of at-risk medical condition in the pre-period (*n*, %)		
No history of at-risk or high-risk medical condition	1 539 526	39.90
History of any at-risk medical condition	2 178 167	56.45
Chronic diseases		
Diabetes mellitus	486 205	12.60
Chronic pulmonary disease (incl. asthma)	1 039 525	26.94
Chronic heart disease	1 576 945	40.87
Neurological disorders	128 109	3.32
Any high-risk medical condition	728 198	18.87
Cancer	247 337	6.41
Cerebrospinal fluid leak	951	0.02
Chronic renal disease	200 377	5.19
Cochlear implant	5837	0.15
Functional or anatomic asplenia, sickle cell disease/other hemaglobinopathy, congenital or acquired asplenia, splenic dysfunction, splenectomy	17 887	0.46
HIV infection	4008	0.10
Immuno-compromising diseases	113 710	2.95
Organ transplant	20 492	0.53
Chronic liver disease	202 125	5.24
Autoimmune disease	100 505	2.60

### Incidence rates

Over the total study period (2016–2019), there were 171 175 episodes of ACP and 1053 episodes of IPD. Rates of ACP and IPD per 100 000 PY are displayed in Table 3 below.

**Table 3.** Incidence rates of ACP and IPD in the overall study population and in each stratification

	ACP		IPD	
	*N* = 171 175		*N* = 1053	
Overall number of episodes	Rate per 100 000 PY	95% CI	Rate per 100 000 PY	95% CI
Overall	1345.43	1339.06–1351.81	8.25	7.76–8.77
Year				
2016	1352.33	1339.86–1364.89	8.01	7.08–9.03
2017	1381.83	1369.06–1394.68	7.57	6.65–8.58
2018	1386.47	1373.52–1399.51	9.52	8.48–10.66
2019	1237.52	1225.13–1250.00	7.76	6.81–8.81
Trend test (*P* value)^a^	1.00	–	1.00	–
Age at study entry				
16–49	512.89	507.49–518.33	1.69	1.40–2.03
50–59	1027.30	1014.59–1040.14	8.76	7.62–10.01
60–69	1675.53	1655.38–1695.85	14.47	12.65–16.47
≥70	4286.49	4257.77–4315.35	24.77	22.64–27.05
Sex				
Male	1442.63	1433.20–1452.11	9.11	8.37–9.89
Female	1252.79	1244.21–1261.42	7.44	6.79–8.13
Presence of medical condition in the pre-period				
At-risk	1397.33	1387.32–1407.39	7.35	6.64–8.12
High-risk	3202.23	3179.36–3225.23	22.48	20.61–24.48
None	426.76	421.08–432.51	2.56	2.14–3.04

ACP, all-cause pneumonia; CI, confidence interval; IPD, invasive pneumococcal disease; PY, person-year.

a Mann–Kendall test for trend.

Throughout the study period, the overall incidence rate of ACP was 1345.43 (95% CI 1339.06–1351.81) episodes per 100 000 PY, with rates increasing with age. Rates of ACP increased slightly over the study period, for all age groups, except for in 2019 where there was a slight decrease. However, the Mann–Kendall test indicated no significant time trends for incidence for the total study population over the study period (*P* = 1.00). The overall incidence rate of ACP was higher in the high-risk and at-risk groups, in comparison to the healthy group.

The overall incidence rate of IPD was 8.25 (95% CI 7.76–8.77) episodes per 100 000 PY. Rates increased with age and were higher in the high-risk and at-risk groups, in comparison to the healthy group. The Mann–Kendall test indicated no significant time trends for IPD incidence over the study period for the overall study population (*P* = 1.00). Incidence rate ratios for the at-risk and high-risk group *vs.* the healthy group were 3.27 (95% CI 3.23–3.32) and 7.50 (95% CI 7.39–7.62), respectively, for ACP, and 2.87 (95% CI 2.68–3.07) and 8.79 (95% CI 8.59–8.98) for IPD (data not presented).

In the overall study population, high-risk individuals with ACP were more often treated as inpatients, while healthy individuals were more likely to be treated as outpatients (S2 Table S1). Incidence rates of ACP for the at-risk population were similar for both the inpatient and outpatient setting. For the 16–49 and 50–59 year old age groups, ACP was more frequently treated in the outpatient setting, irrespective of presence of risk conditions. Whereas, individuals aged 70 and over were more often treated as inpatients, even for healthy individuals without risk conditions. For the 60–69 age group, ACP incidence rates were similar in the inpatient *vs.* outpatient setting for all risk groups. For males, incidence rates of ACP were greater in the inpatient *vs.* outpatient setting overall, and for individuals aged over 60.

### Fatality rates

Throughout the study period (2016–2019), there were 15 983 deaths for hospitalised cases of ACP and 164 deaths for hospitalised cases of IPD. Fatality rates for ACP and IPD are displayed in Table 4, overall and for each stratification.

**Table 4.** Fatality rates for hospitalised cases of ACP and IPD in the overall study population and in each stratification

	ACP		IPD	
	*N* = 15 983		*N* = 164	
Overall number of deaths	Rate per 100 cases	95% CI	Rate per 100 cases	95% CI
Overall	17.22	16.98–17.47	16.89	14.66–19.38
Year				
2016	16.92	16.45–17.39	15.60	11.63–20.61
2017	17.30	16.83–17.78	16.59	12.28–22.03
2018	17.82	17.33–18.31	18.73	14.61–23.68
2019	16.75	16.25–17.26	16.13	11.83–21.60
Trend test (*P* value)^a^	1.00	–	0.75	–
Age at study entry				
16–49	4.42	3.98–4.91	8.74	4.67–15.78
50–59	10.95	10.32–11.62	13.16	9.07–18.70
60–69	14.10	13.54–14.68	12.50	8.73–17.58
≥70	20.36	20.04–20.67	22.29	18.74–26.31
Sex				
Male	17.64	17.31–17.97	18.10	15.07–21.58
Female	16.70	16.34–17.06	15.40	12.31–19.10
Presence of medical condition in the pre-period				
At-risk	15.32	14.96–15.69	14.52	11.30–18.46
High-risk	19.60	19.25–19.95	18.84	15.60–22.57
None	10.17	9.47–10.92	16.38	10.74–24.17

ACP, all-cause pneumonia; CI, confidence interval; IPD, invasive pneumococcal disease.

a Mann–Kendall test for trend.

Overall, the fatality rate of ACP was 17.22 (95% CI 16.98–17.47) deaths per 100 hospitalised cases of ACP. The fatality rate of IPD overall was 16.89 (95% CI 14.66–19.38) deaths per 100 hospitalised cases. Fatality rates remained steady throughout the entire study period, for both ACP and IPD (*P* = 1.00 and *P* = 0.75, respectively). Fatality rates for both ACP and IPD increased with age and were highest in the high-risk, followed by the at-risk group, in comparison to healthy individuals.

Fatality rate ratios for the at-risk and high-risk group *vs.* the healthy group were 1.51 (95% CI 1.40–1.62) and 1.61 (95% CI 1.50–1.73), respectively, for ACP, and 0.88 (95% CI 0.55–1.1430) and 1.15 (95% CI 0.73–1.81) for IPD (data not presented).

### Healthcare resource utilisation and cost

Over the study period, there were a total of 3795 outpatient visits per 1000 PY (95% CI 3775–3815), 2928 (95% CI 2910–2946) inpatient hospital admissions per 1000 PY and 3726 (95% CI 3706–3746) prescriptions per 1000 PY associated with ACP in patients with at least one ACP episode. In total, there were 1580 (95% CI 1404–1772) per 1000 PY outpatient visits, 5156 (95% CI 4834–5493) inpatient hospital admissions per 1000 PY and 3482 (95% CI 2581–4126) prescriptions per 1000 PY associated with IPD in patients with at least one episode of IPD. Overall HCRU over the total study period is presented in Table 5 for ACP and IPD. Costs per episode and overall are presented for each resource in Table 6.

**Table 5.** HCRU associated with ACP and IPD in patients with at least one ACP or IPD episode, by age (2016–2019)

	ACP					IPD				
	Overall	16–49	50–59	60–69	≥70	Overall	16–49	50–59	60–69	≥70
	*N* = 146 225	*N* = 31 166	*N* = 21 594	*N* = 22 306	*N* = 79 258	*N* = 965	*N* = 103	*N* = 185	*N* = 210	*N* = 467
Outpatients										
Number of patients (*n*, %)	78 737 (53.85%)	26 601 (85.35%)	15 476 (71.67%)	11 879 (53%)	24 781 (35%)	176 (18.24%)	31 (30.10%)	33 (18%)	31 (15%)	81 (17%)
Visit rate (per 1000 PY, 95% CI)	3795 (3775–3815)	5638 (5585–5691)	4753 (4697–4811)	3723 (3673–3773)	2609 (2584–2634)	1580 (1404–1772)	2354 (1763–3079)	1528 (1154–1985)	1160 (855–1538)	1602 (1344–1895)
Number of prescriptions (*n*)	132 493	43 255	26 219	20 899	42 120	369	74	83	69	143
Prescription rate (per 1000 PY, 95% CI)	3726 (3706–3746)	5516 (5464–5568)	4659 (4603–4716)	3664 (3614–3714)	2570 (2546–2595)	3482 (2581–4126)	3287 (2211–3523)	2265 (1804–2808)	1667 (1297–2110)	1685 (1420–1985)
Inpatients										
Number of patients (*n*, %)	82 255 (56.25%)	7014 (22.51%)	8055 (37.30%)	12 709 (57%)	54 477 (77%)	897 (92.95%)	92 (89.32%)	171 (92%)	200 (95%)	434 (93%)
Number of hospital admissions	104 127	8149	9917	16 245	69 816	956	103	185	211	457
Hospital admission rate (per 1000 PY, 95% CI)	2928 (2910–2946)	1039 (1017–1062)	1762 (1728–1797)	2848 (2804–2892)	4261 (4229–4292)	5156 (4834–5493)	4575 (3734–5549)	5049 (4347–5831)	5098 (4433–5834)	5384 (4902–5902)
Length of stay (days)										
Mean (Min, max)	16 (1–437)	14 (1–298)	18 (1–406)	18 (1–437)	16 (1–291)	18 (1–169)	19 (1–106)	19 (1–169)	18 (1–106)	17 (1–92)

ACP, all-cause pneumonia; CI, confidence interval; IPD, invasive pneumococcal disease; PY, person-year.

**Table 6.** Healthcare cost associated with ACP and IPD in patients with at least one ACP or IPD episode, by risk groups (2016–2019)

	ACP				IPD			
	Overall	High-risk	At-risk	Healthy	Overall	High-risk	At-risk	Healthy
Number of patients	146 225	61 547	64 534	20 144	965	486	364	115
Outpatients								
Visit rate (rate per 1000 PY, 95% CI)	3795 (3775–3815)	3180 (3152–3209)	4028 (3997–4060)	4976 (4913–5040)	1580 (1404–1772)	1615 (1369–1892)	1465 (1191–1784)	1774 (1273–2407)
Total visit cost, €	6 884 025	2 989 113	3 036 097	858 814	18 946	9406	7764	1776
Visit cost per episode, €, (95% CI)	40 (40–41)	40 (39–41)	41 (40–41)	40 (9–41)	18 (13–23)	18 (13–23)	20 (9–30)	14 (3–25)
Total outpatient cost, including visits and prescriptions, €	9 464 319	3 995 777	4 205 183	1 263 360	31 034	15 886	11 533	3615
Outpatient cost per episode, €, (95% CI)	55 (55–56)	53 (52–54)	56 (56–57)	59 (8–60)	29 (22–37)	30 (19–41)	29 (17–41)	28 (14–42)
Inpatients								
Hospital admission (rate per 1000 PY, 95% CI)	2928 (2910–2946)	3701 (3670–3732)	2599 (2574–2625)	1563 (1527–1599)	5156 (4834–5493)	5024 (4583–5497)	5417 (4876–6001)	4933 (4069–5926)
Total hospital cost, €	239 426 680	127 976 977	94 020 761	17 428 941	8 471 725	4 360 150	2 838 932	1 272 643
Hospital cost per episode, €, (95% CI)	1399 (1370–1427)	1706 (1659–1752)	1259 (1218–1301)	811 (747–875)	8045 (7092–8999)	8227 (6848–9606)	7205 (5827–8584)	9865 (6536–13 195)
Total inpatient and outpatient cost (€)	248 891 000	131 972 754	98 225 945	18 692 301	8 502 759	4 376 036	2 850 465	1 276 258
Total cost per episode (€, 95% CI)	1454 (1426–1482)	1759 (1712–1806)	1316 (1275–1357)	870 (805–934)	8075 (7121–9028)	8257 (6878–9635)	7235 (5857–8612)	9893 (6565–13 222)

ACP, all-cause pneumonia; CI, confidence interval; IPD, invasive pneumococcal disease; PY, person-year.

Rate of outpatient admissions was higher for ACP than IPD. Pharmacy cost per episode and rate of prescriptions in the outpatient setting were also higher for ACP. However, rate of inpatient hospital admissions and cost of hospital admissions per episode were significantly higher for IPD than ACP.

For ACP, rate of inpatient admissions increased with age ranging from 1039 per 1000 PY (95% CI 1017–1062) for 16–49 year olds to 4261 (95% CI 4229–4292) per 1000 PY for individuals aged 70 and over. For IPD patients, the rate of inpatient admissions remained relatively steady across age-groups.

For both ACP and IPD, rate of outpatient admissions decreased with age ranging for ACP patients from 5638 (95% CI 5585–5691) per 1000 PY in 16–49 year olds to 2609 (95% CI 2584–2634) per 1000 PY for individuals aged 70 and over. Similarly, for IPD patients the rate ranged from 2354 (95% CI 1763–3079) per 1000 PY in 16–49 year olds to 1602 (95% CI 1344–1895) per 1000 PY for individuals aged 70 and over; although there was a slight increase in the individuals aged ≥70 compared to 60–69 year olds.

For ACP, rate of outpatient admissions was higher in the at-risk group compared to the high-risk group; 4028 (95% CI 3997–4060) per 1000 PY *vs.* 3180 (95% CI 3152–3209) per 1000 PY for the at-risk *vs.* high-risk group, respectively (Table 6). Rate of hospital admissions was greatest for the high-risk group; 2599 (95% CI 2574–2625) per 1000 PY for the at-risk group and 3701 (95% CI 3670–3732) per 1000 PY for the high-risk group. Total hospital cost per episode was also greatest for the high-risk group; €1259 (95% CI €1218–1301) for the at-risk and €1706 (95% CI 1659–1752) for the high-risk group, respectively. For IPD, rate of outpatient visits, hospital admissions and cost per episode did not vary significantly by risk group (Table 6).

Over the entire study period, the mean total inpatient and outpatient direct medical costs for IPD and ACP per episode were estimated to be €8075 (95% CI 7121–9028) and €1454 (95% CI 1426 −1482) respectively (Table 6). The aggregate direct medical costs for IPD and ACP were estimated to be €8.5 million and €248.9 million respectively. Total cost per episode increased with age for ACP: from €680 (95% CI 636–725) in the 16–49 age group to €1779 (95% CI 1743–1815) in those ≥70 (data not presented). In contrast, cost per episode remained steady for those with IPD.

## Discussion

The results of the present study provide estimates for clinical and economic burden of ACP and IPD in Germany. Overall incidence rates of ACP and IPD over the study period were 1345.43 (95% CI 1339.06–1351.81) episodes per 100 000 PY and 8.25 (95% CI 7.76–8.77) episodes per 100 000 PY, respectively. Overall fatality rates for ACP and IPD were 17.22 (95% CI 16.98–17.47) and 16.89 (95% CI 14.66–19.38) deaths per 100 hospitalised cases, respectively. For both ACP and IPD, incidence and fatality rates increased with age and risk conditions. Mann–Kendall tests indicated no significant time trends for incidence of ACP or IPD for the overall study population over the study period (2016–2019, *P* = 1.00). Fatality rates for both ACP and IPD also remained steady over the study period (*P* = 1.00 and 0.75, respectively). Incidence rate ratios for ACP and IPD incidence were almost eight-fold in people with high-risk conditions compared to the healthy group and fatality rates were almost two-times higher in this group compared to healthy in patients with ACP. In the overall population, high-risk individuals with ACP were more often treated in the inpatient setting, while healthy individuals were more likely to be treated in the outpatient setting. For the 16–49 and 50–59 year old age groups, ACP was more frequently treated in the outpatient setting, irrespective of risk group. Whereas individuals aged 70 and over were more often treated in the inpatient setting, irrespective of risk group. ACP and IPD in Germany were associated with high direct economic costs over the study period and were highest amongst high-risk groups.

This study is the first to quantify both the clinical and economic burden of both ACP and IPD in Germany in adults. Our results are consistent with prior estimates of annual incidence rates of PD in adults throughout Europe – estimated to range from 1.1 to 14 per 1000 PY and remaining relatively consistent in the last few decades [5, 10, 26, 27]. Previous studies exploring the incidence of PD or the burden in Germany have predominantly focussed on specific subpopulations. A previous study by Pelton and colleagues, also using the InGef database (study period 2009–2012) compared the incidence rates of ACP in children and adults with chronic medical conditions [7]. In alignment with this study, the reported rates of ACP (same case definition) were higher among adults with high-risk or at-risk conditions *vs.* healthy counterparts. Consistent with other studies of PD in Germany, Pelton and colleagues also reported increasing incidence of ACP with increasing age within each risk group (at-risk, high-risk and healthy), with relatively stable rate ratios, suggesting that the relative increase in disease risk with age is similar across risk groups [7, 28, 29]. Indeed, it is well established that the incidence of PD in adults increases with age and presence of risk conditions, whereby aging itself increases the risk of PD due to immunosenescence, multimorbidity (including an increase in risk conditions) and frailty [7, 28–31]. In the study by Pelton *et al*., the incidence rate of ACP in younger adults (aged 18–49) at high-risk of PD due to underlying conditions was not dissimilar to the incidence rate of ACP in healthy older adults (aged over 60); as has been demonstrated in other studies in Germany [29, 32].

Where to treat a patient with pneumonia is arguably the most important management decision for determining both clinical outcome and cost [33]. In our study, ACP was more frequently treated in the outpatient setting for the 16–49 and 50–59 year old age groups, irrespective of presence of risk conditions. Whereas, individuals aged 70 and over were more often treated in the inpatient setting, even for healthy individuals without risk conditions. This is unsurprising given that German recommendations for the treatment of pneumonia indicate severity risk stratification to determine which patients may be amenable to treatment in outpatient care; whereby age and comorbidity are important factors associated with PD severity and mortality, older age associated with increasing multimorbidity [33–35]. Recent evidence pleads for increased consideration of comorbidities in determining management of PD [36, 37]. It has been suggested that the stacking of comorbidities and type of comorbidity may to an extent predict pneumonia severity independently of age, with the exception of nonagenarians where mortality increase seems to be more related to the age itself rather than increasing comorbidity [36, 37].

An additional study conducted in InGef by Schmedt and colleagues investigated incidence rates of community-acquired pneumonia (CAP) in individuals with cancer aged 18 years or over and associated HCRU, during the study period 2011–2015 [38]. CAP cases were defined as patients with a primary hospital diagnosis of pneumonia (ICD-10-GM codes, J12–J18) or a secondary hospital diagnosis in combination with a hospital admission diagnosis of pneumonia. Over the total study period, the incidence rate of CAP was 451.8 (95% CI 448.1–455.4) episodes per 100 000 PY. This is significantly lower than the rate of ACP reported in the present study. This may be explained by the difference in coding of ACP and the definition of the study population. In our study we included all causes of pneumonia (ICD-10-GM J10.0, J11.0, J12–J18) including viral pneumonia (ICD-10-GM J10.0 and J11.0) which may contribute significantly to the substantial burden of ACP [39].

A recent study by Van der Linden and colleagues explored incidence of IPD over time as reported to the German National Reference Center for Streptococci, from 2003–2018. Cases of IPD per 100 000 population of Germany were shown to be substantial and increased over time for all adult age groups (16–49, 50–59, 60–75, >75), most dramatically in those aged 60 and over: from 1.64 in 2003–2006 (before the universal vaccination recommendation for children was introduced) to 10.08 cases per 100 000 population in 2017/2018 [40]. The authors suggest that this is due to both the aging population, and a rise in non-vaccinated pneumococcal serotypes, particularly serotype 3. This trend did not change with the switch to PCV13 childhood vaccination in 2009. This highlights the need for expanded pneumococcal serotype coverage with future vaccines.

Similar to the study by Van der Linden and colleagues, in our study, burden of IPD remained substantial over the time period 2016–2019. The lack of trends observed over the study period could be as a result of reaching the plateau phase in the incidence of pneumococcal disease due to serotype replacement, and no substantial changes in PCV recommendations. Continued surveillance of PD incidence following the introduction of novel, higher valency PCVs will be warranted – with two new PCVs, PCV15 and PCV20, recently licensed in the US for prevention of IPD in adults [41]. Although these novel PCVs expand protection against PD, serotype replacement will be a key factor for consideration in their economic evaluation, with 100 distinct pneumococcal serotypes currently identified [42].

Studies since the introduction of the PCV13 in other countries, for example in the UK [43] or US [44], have shown a decline in IPD over time in adults aged over 65, attributing it to herd-protection after the introduction of the PCV13 vaccine in children. It cannot be assumed that such declines will be mirrored in other Western countries. Furthermore, these studies were conducted in earlier time-periods, perhaps before the changes in serotype distribution and compensatory increase in non-vaccinated pneumococcal serotypes. A recent study by Hanquet *et al*., reported an overall decrease in cases of IPD in 10 European countries across the total study period from 2009–2015, but found an increase in IPD cases in the final year of their study [45]. According to surveillance data, SURVSTAT, from the Robert Koch Institute, the number of observed cases of IPD in Germany for adults in all age groups increased from 2010 to 2016 - from an overall estimated incidence (all age groups) of 0.45 per 100 000 population in 2010 to 0.78 per 100 000 population in 2016 [46, 47]. Of note, reported cases in the elderly (>60) nearly doubled.

PD-related fatality rates for Germany for the years 2001–2014 have previously been described utilising data from the World Health Organization European detailed mortality database (WHO EDMD) [8]. Fatality rates decreased by 26.8% and 29.8% for males and females respectively. In the present study, fatality rates were instead defined as deaths in hospitalised cases of ACP/IPD; with no significant trends identified over the study period (2016–2019).

Healthcare cost of pneumonia has previously been estimated in adults in Germany for 2003. A prospective study in 22 hospitals found that the median cost of a hospitalised patient was €1201 [48]; in alignment with the present study, whereby cost per episode was estimated as €1399 (95% CI 1370–1427) for ACP. Both in the present study and in previous studies, incidence of ACP/IPD throughout Europe has been shown to increase with age and in those with a higher number of comorbid conditions [7, 27]. With an aging European population, it is anticipated that these costs will increase in the future [8, 10, 11].

In August 2016, a new pneumococcal vaccination recommendation for Germany was published by STIKO: PPSV23 recommended for all adults aged 60 and over and all individuals aged ≥16 with at least 1 chronic disease not associated with immune suppression. A novel vaccination reimbursement directive was also adopted in May 2017 in Germany, defining obligatory reimbursement and funding of these pneumococcal vaccination recommendations. This may encourage actioning of such recommendations and improvements in previously reported low vaccination coverage rates (VCR) in Germany [49–51] – likely also contributing to the maintained substantial clinical and economic burden of ACP and IPD in the present study. It is important that incidence rates of PD and VCR are continually monitored across Germany to assess the impact of such vaccination schedules and incentives.

There were several limitations to this study. The first relates to the representativeness of the InGef database of the German population. Previous studies however have demonstrated a good overall accordance of the InGef research database and the German population in terms of measures of morbidity, mortality and drug usage [23]. Unfortunately, information on serotype distribution was not available in the InGef database. An understanding of prevalent and emerging serotypes will be critical when considering the development/ introduction of novel pneumococcal vaccines. Information on date of death (during hospital stays or outside of hospital) is available in the InGef research database, however cause of death is not recorded. There may be more bias for mortality outside of hospital as it may occur due to reasons other than pneumococcal disease. Thus, we decided to only use mortality at hospital discharge. This may have underestimated the case fatality rate of pneumococcal disease. Indeed, a recent study utilising the InGef database estimated all-cause mortality after hospitalised CAP in 2015 as 18.5% in-hospital, 22.9% at 30-days and 44.5% at one year [29]. Similarly, in the outpatient data, only yearly quarter in which diagnoses were made were available, not exact diagnosis dates. Antibiotic prescriptions and diagnostic tests during the quarters with ACP/IPD diagnoses were assumed to be related to ACP/IPD to assign exact diagnosis dates.

There may also be a risk of misclassification bias due to coding inaccuracies since medical conditions were identified based on existing records. Misclassification bias due to under recoding of pneumonia is believed however to be non-differential with respect to comparison groups. In addition, prior validation studies suggest that ICD-based claim codes have good sensitivity and specificity for the identification of pneumonia cases [52, 53].

The purpose of this study is to report the burden of PD potentially preventable by pneumococcal vaccination, and therefore focussed on the unvaccinated population. ACP was chosen as a proxy for pneumococcal pneumonia, as coding of pneumococcal pneumonia within administrative healthcare databases would underestimate disease burden [7, 24, 25]. Nevertheless, our study outcome of ACP includes diagnoses such as viral pneumonia, and may therefore overestimate the burden of vaccine preventable PD.

Of note, evidence has suggested that the effectiveness of PCVs may be age-dependent; with particularly reduced vaccine efficacy suggested for those aged 85 years and older (although study power was limited) [54, 55]. Therefore, our study may be limited by our upper-age stratification of 70 years and older.

Finally, the rates estimated in this study were not adjusted for any covariates such as age, sex or chronic diseases as the aim of this study was to describe the burden in different subpopulations and therefore are presented as crude rates and their CIs. However, relevant confounding factors remain. For example, it is well established that incidence of PD increases with age, and that prevalence of risk conditions predisposing to PD also increase with age. Therefore, the healthy, at-risk and high-risk populations have different age distributions. Although beyond the scope of the present study, Pelton and colleagues previously explored incidence rates of ACP by age and risk for the population of the InGef database, as discussed above, and demonstrated increasing incidence of ACP with increasing age within each risk group (at-risk, high-risk and healthy), with relatively stable rate ratios. This suggests that the relative increase in disease risk with age is similar across all three risk groups [7]. Future studies would be needed to further explore the age/risk relationship in terms of HCRU and cost of PD.

## Conclusion

In summary, this study demonstrates that mortality and morbidity due to ACP and IPD remains high in the German adult population and increases with age and presence of comorbid risk conditions. The clinical and economic burden of IPD and ACP among German adults is substantial regardless of age. Continued surveillance of VCR, serotype distribution, and the clinical and economic burden of ACP and IPD is recommended. Such data will be critical to assess the impact of current vaccination schedules and to estimate the potential benefit of future PCVs.

## Data Availability

The data that support the findings of this study are stored within the Institute for Applied Health Research Berlin GmbH (InGef, www.InGef.de). Restrictions apply to the availability of these data, and they are not publicly available. Access to patient-level data is not possible and all analyses must be conducted by InGef. Requests for bespoke analyses/ aggregate results are reviewed and approved by InGef.

## Supplementary material

For supplementary material accompanying this paper visit http://doi.org/10.1017/S0950268822001182. click here to view supplementary material
