# Supplementary material for: Clinical and economic burden of pneumococcal disease among individuals aged 16 years and older in Germany
Source: Epidemiol Infect. 2022 Nov 8;150:e204. doi: 10.1017/S0950268822001182 (PMC9987016; doi:10.1017/S0950268822001182)
Supplement: Supplementary file 1 [file S0950268822001182sup001.docx]

# Supplementary Information

**S1 Code lists**

**Table 1. All-cause pneumonia ICD-10-GM code list**

| **Category and ICD-10-GM** |
| --- |
| *All-cause pneumonia*  J13, J15.9 + B95.3, J18.0 + B95.3, J18.1 + B95.3, J18.8 + B95.3, J18.9 + B95.3, J15.9, J18.0, J18.1, J18.8, J18.9, J10.0, J11.0, J12, J14, A48.1, J15.0, J15.1, J15.2, J15.3, J15.4, J15.5, J15.6, J15.7, J15.8, J16, A22.1, A37, B25.0, B44.0, J17, J18 |

**Table 2. IPD ICD-10-GM code list**

| **Category and ICD-10-GM** |
| --- |
| *Bacteremia*  A40.3, A40.9 + B95.3, A41.9 + B95.3, A49.9 + B95.3 |
| *Meningitis*  G00.1, G00.2 + B95.3, G00.9 + B95.3, G03.9 + B95.3 |
| *Bacteremic pneumonia*  A22.1 + A40.3, A22.1 + A40.9 + B95.3, A22.1 + A41.9 + B95.3, A22.1 + A49.9 + B95.3, A37 + A40.3, A37 + A40.9 + B95.3, A37 + A41.9 + B95.3, A37 + A49.9 + B95.3, A48.1 + A40.3, A48.1 + A40.9 + B95.3, A48.1 + A41.9 + B95.3, A48.1 + A49.9 + B95.3, B25.0 + A40.3, B25.0 + A40.9 + B95.3, B25.0 + A41.9 + B95.3, B25.0 + A49.9 + B95.3, B44.0 + A40.3, B44.0 + A40.9 + B95.3, B44.0 + A41.9 + B95.3, B44.0 + A49.9 + B95.3, J09 + A40.3, J09 + A40.9 + B95.3, J09 + A41.9 + B95.3, J09 + A49.9 + B95.3, J10.0 + A40.3, J10.0 + A40.9 + B95.3, J10.0 + A41.9 + B95.3, J10.0 + A49.9 + B95.3, J11.0 + A40.3, J11.0 + A40.9 + B95.3, J11.0 + A41.9 + B95.3, J11.0 + A49.9 + B95.3, J12 + A40.9 + B95.3, J12 + A41.9 + B95.3, J12 + A49.9 + B95.3, J12+ A40.3, J13 + A40.3, J13 + A40.9, J13 + A40.9 + B95.3, J13 + A41.9, J13 + A41.9 + B95.3, J13 + A49.9, J13 + A49.9 + B95.3, J14 + A40.3, J14 + A40.9 + B95.3, J14 + A41.9 + B95.3, J14 + A49.9 + B95.3, J15 + A40.3, J15 + A40.9 + B95.3, J15 + A41.9 + B95.3, J15 + A49.9 + B95.3, J16 + A40.3, J16 + A40.9 + B95.3, J16 + A41.9 + B95.3, J16 + A49.9 + B95.3, J17 + A40.3, J17 + A40.9 + B95.3, J17 + A41.9 + B95.3, J17 + A49.9 + B95.3, J18.0 + A40.3, J18.0 + A40.9 + B95.3, J18.0 + A41.9 + B95.3, J18.0 + A49.9 + B95.3, J18.1 + A40.3, J18.1 + A40.9 + B95.3, J18.1 + A41.9 + B95.3, J18.1 + A49.9 + B95.3, J18.8 + A40.3, J18.8 + A40.9 + B95.3, J18.8 + A41.9 + B95.3, J18.8 + A49.9 + B95.3, J18.9 + A40.3, J18.9 + A40.9 + B95.3, J18.9 + A41.9 + B95.3, J18.9 + A49.9 + B95.3, J85.1 + B95.3, J86.0 + B95.3, J86.9 + B95.3, A22.1 + A40.9 , A22.1 + A41.9, A22.1 + A49.9, A37 + A40.9, A37 + A41.9, A37 + A49.9, A48.1 + A40.9, A48.1 + A41.9, A48.1 + A49.9, B25.0 + A40.9 , B25.0 + A41.9, B25.0 + A49.9, B44.0 + A40.9, B44.0 + A41.9, B44.0 + A49.9, J09 + A40.9, J10.0 + A40.9, J10.0 + A41.9, J10.0 + A49.9, J11.0 + A40.9, J11.0 + A41.9, J11.0 + A49.9, J12 + A41.9, J12 + A49.9, J13 + A40.9, J14 + A40.9, J14 + A41.9, J14 + A49.9, J15 + A40.9, J15 + A41.9, J15 + A49.9, J16 + A40.9, J16 + A41.9, J16 + A49.9, J17 + A40.9, J17 + A41.9, J17 + A49.9, J18.0 + A40.9, J18.0 + A41.9, J18.0 + A49.9, J18.2 + A40.9, J18.2 + A41.9, J18.2 + A49.9, J18.8 + A40.9, J18.8 + A41.9, J18.8 + A49.9, J18.9 + A40.9, J18.9 + A41.9, J18.9 + A49.9 |
| *Other IPD*  I30.1 + B95.3, I33.0 + B95.3, I33.9 + B95.3, K65.0 + B95.3, K65.2 + B95.3, K65.8 + B95.3, K65.8 + B95.3, K65.9 + B95.3, M00.0 + B95.3, M00.1, M00.1 + B95.3, M00.2 + B95.3, M00.8 + B95.3 , M00.9 + B95.3, M86.1 + B95.3, M86.2 + B95.3, M86.9 + B95.3 |

**S2 Additional tables**

**Table 1. Incidence rates of ACP by inpatient and outpatient setting**

|  | **ACP inpatients** | | **ACP outpatients** | |
| --- | --- | --- | --- | --- |
| **Overall number of episodes** | **N= 171175** | | **N= 1053** | |
|  | **Rate per 100000 PY** | **95% CI** | **Rate per 100000 PY** | **95% CI** |
| **Age group** |  |  |  |  |
| 16-49 | 111.41 | 108.90-113.96 | 401.28 | 396.51-406.10 |
| 50-59 | 365.55 | 357.99-373.23 | 660.68 | 650.50-670.98 |
| 60-69 | 914.42 | 899.57-929.45 | 758.09 | 744.57-771.79 |
| ≥70 | 3152.67 | 3128.08-3177.40 | 1119.46 | 1104.85-1134.22 |
| **Sex** |  |  |  |  |
| *Overall* |  |  |  |  |
| Male | 843.81 | 836.61-851.06 | 596.70 | 590.65-602.81 |
| Female | 632.76 | 626.67-638.89 | 618.39 | 612.37-624.46 |
| *Aged 16-49* |  |  |  |  |
| Male | 125.87 | 122.13-129.69 | 387.08 | 380.51-393.74 |
| Female | 96.51 | 93.20-99.92 | 415.91 | 408.99-422.91 |
| *Aged 50-59* |  |  |  |  |
| Male | 463.66 | 451.65-475.91 | 669.41 | 654.96-684.10 |
| Female | 266.94 | 257.83-276.30 | 651.90 | 637.60-666.44 |
| *Aged 60-69* |  |  |  |  |
| Male | 1209.57 | 1185.00-1234.51 | 774.41 | 754.79-794.41 |
| Female | 639.19 | 621.98-656.76 | 742.85 | 724.28-761.78 |
| *Aged ≥70* |  |  |  |  |
| Male | 4065.04 | 4021.48-4108.94 | 1194.50 | 1170.99-1218.35 |
| Female | 2517.60 | 2489.01-2546.44 | 1067.11 | 1048.54-1085.92 |
| **Presence of medical condition in the pre-period** | | | |  |
| *Overall* | | | |  |
| At-risk | 694.94 | 687.90-702.04 | 700.32 | 693.25-707.45 |
| High-risk | 2120.34 | 2101.76-2139.05 | 1072.40 | 1059.21-1085.72 |
| None | 133.89 | 130.72-137.13 | 292.70 | 287.99-297.46 |
| *Aged 16-49* | | | | |
| At-risk | 134.33 | 129.67-139.12 | 546.17 | 536.73-555.74 |
| High-risk | 301.19 | 287.74-315.10 | 656.95 | 637.01-677.35 |
| None | 65.76 | 63.20-68.40 | 269.33 | 264.12-274.62 |
| *Aged 50-59* | | | | |
| At-risk | 353.34 | 342.68-364.25 | 756.76 | 741.11-772.65 |
| High-risk | 725.22 | 700.66-750.42 | 943.85 | 915.79-972.54 |
| None | 179.27 | 170.11-188.80 | 358.70 | 345.70-372.08 |
| *Aged 60-69* | | | | |
| At-risk | 747.01 | 728.74-765.62 | 736.77 | 718.63-755.25 |
| High-risk | 1600.50 | 1563.04-1638.62 | 1066.99 | 1036.45-1098.19 |
| None | 392.86 | 370.64-416.07 | 368.31 | 346.80-390.80 |
| *Aged ≥70* | | | | |
| At-risk | 2377.32 | 2347.05-2407.88 | 964.50 | 945.28-984.02 |
| High-risk | 4511.18 | 4465.74-4556.95 | 1454.18 | 1428.50-1480.21 |
| None | 984.37 | 937.66-1032.80 | 368.92 | 340.56-399.01 |

ACP: all-cause pneumonia; CI: confidence interval; IPD: invasive pneumococcal disease; PY: person-year.

* Mann-Kendall test for trend
